# Temporal trend of tracheostomy in patients hospitalized in the Brazilian National Unified Health System from 2011 to 2020

**DOI:** 10.1590/0100-6991e-20223373-en

**Published:** 2022-08-25

**Authors:** LUIZA CASCAES NAZARIO, FLÁVIO RICARDO LIBERALI MAGAJEWSKI, NATALIA DAL PIZZOL, MATHEUS HENRIQUE DA SILVA SALOTI, LEONARDO KFOURI MEDEIROS

**Affiliations:** 1 - Universidade do Sul de Santa Catarina, Medicina - Tubarão - SC - Brasil

**Keywords:** Tracheostomy, Unified Health System, Respiratory Tract Diseases, Traqueostomia, Sistema Único de Saúde, Doenças do Sistema Respiratório

## Abstract

**Objective:**

to analyze the temporal trend in the tracheostomy use in patients hospitalized by the Sistema Único de Saúde in Brazil from 2011 to 2020.

**Methods:**

ecological observational study with a quantitative approach and including the Brazilian population aged 20 or over that were admitted by Sistema único de Saúde and had a record of performing the tracheostomy procedure at any time during hospitalization.

**Results:**

113.569.570 Hospitalizations studied were identified 172.456 tracheostomies realized in Brazil (0,15%). The average tax of this procedure showed a downward trend during the study procedure. The highest tracheostomy rate was found in the southern region, and the most affected age group was 80 years old or more. The average rate of tracheostomy in males was 1.8 times higher than in females. The average mortality and lethality rates of admissions with tracheostomy were 3.36 and 28.57% in the period but showed a tendency to decrease in the period studied. The main causes associated with the performance of tracheostomy were respiratory, oncological, and external causes. Respiratory causes contributed to 73% of the total procedures performed in the analyzed period.

**Conclusion:**

the average mortality and lethality rates of hospitalizations with tracheostomy in Brazil were 3.36 and 28.57%, but showed a downward trend in the period.

## RESUMO

**Objetivo:**

analisar a tendência temporal da utilização da traqueostomia em pacientes hospitalizados pelo Sistema Único de Saúde no Brasil no período de 2011 a 2020.

**Método:**

estudo observacional de tipo ecológico, com abordagem quantitativa, e incluiu a população brasileira com 20 anos ou mais que, internadas pelo Sistema Único de Saúde, tiveram registro de realização do procedimento de traqueostomia em qualquer momento da hospitalização.

**Resultados:**

das 113.569.570 hospitalizações estudadas, foram identificadas 172.456 traqueostomias realizadas no Brasil (0,15%). A taxa média de realização deste procedimento apresentou tendência de queda no período estudado. A maior taxa média de traqueostomia foi encontrada na Região Sul, e a faixa etária mais afetada foi a dos 80 anos ou mais. A taxa média de traqueostomia no sexo masculino foi de 1,8 vezes maior do que no sexo feminino. As principais causas associadas à realização de traqueostomia foram as patologias respiratórias, oncológicas e decorrentes de causas externas, sendo que as causas respiratórias contribuíram com 73% do total de procedimentos estudados.

**Conclusões:**

as taxas médias de mortalidade e letalidade das internações com traqueostomia no Brasil foram de 3,36 e 28,57%, mas apresentaram tendência de redução no período.

## INTRODUCTION

Tracheostomy is a surgical procedure, very common in critically ill patients, consisting of opening the anterior wall of the trachea, creating a communication between the respiratory tree and the external environment, where a cannula is inserted to facilitate breathing¹. The advantages of tracheostomy include the reduction of anatomical dead space and airway resistance, which facilitates respiratory mechanics and, in critically ill patients, improves airway maintenance with more safety and comfort².

There are records of this procedure being performed since ancient civilizations, when facing airway obstruction, usually of a traumatic nature. Currently, it is more used in prolonged mechanical ventilation than for upper airway clearance^4^.

Tracheostomy can be performed in several ways. The open technique is usually performed in the operating room, and requires patient transport and anesthesia. However, moving the patient, who is usually in a critical condition, can bring complications and require greater support from the team^5^ ^,^ ^6^. The other option is percutaneous dilation, a technique created in the 1980s to overcome circumstances that limited the use of the open surgical technique. This procedure is simpler when compared with the open technique, and can be performed at the bedside, with fewer professionals and reduced costs^7^. However, regardless of the technique used, it is essential to individualize each situation to choose the most appropriate method for each patient, and to have a team prepared to solve usual or unexpected problems associated with the procedure^3^.

Normally, indications for tracheostomy are equivalent for both the standard and the percutaneous procedures^3^. The main indications are prolonged mechanical ventilation and difficult or long-term weaning. An example of this is that 10% of patients who are on mechanical ventilation end up undergoing tracheostomy with the aim of weaning from ventilation^8^. Most patients undergoing tracheostomy are admitted to the hospital for causes such as acute respiratory failure, trauma, neuromuscular diseases, and coma^9^. Thus, the main indications for tracheostomy are classically divided into four groups: (1) conditions associated with upper airway obstruction; (2) conditions that prevent alveolar ventilation and require assisted breathing; (3) conditions with excessive airway secretions; (4) and other indications^3^.

The benefits of tracheostomy include less breathing effort, simplification of bronchial hygiene, and greater comfort for the patient^10^. Tracheostomy also implies relative benefits, such as earlier withdrawal from mechanical ventilation, less damage to the patient’s vocal cords, a possible reduction in sedation^11^ , and a reduction in incidents such as accidental extubation and tube occlusion, which are potentially fatal^11^.

Like any other medical procedure, tracheostomy has risks and complications, both early and late. The early ones, which occur in the first days after the procedure, are often associated with the professional’s inexperience and the lack of preventive measures^3^. The main complications at this stage include obstructions, pneumothorax, bleeding, and postoperative infections. Late complications, which appear weeks or even months after the procedure, include tracheal stenosis and malacia, tracheoarterial fistula, and reduced phonation^12^.

In summary, tracheostomy is common, relatively simple, and relevant when well indicated, and used in complex situations where ventilatory support is a critical condition. It is part of the list of possibilities in the treatment of oncology, otorhinolaryngological, neurological, traumatized, and respiratory patients, and is directly related to the management of critically ill patients. However, despite being frequent and associated with numerous clinical situations, it is not a commonly studied topic in the medical literature. For these reasons, we consider the clarification of its frequency, the profile of the patients submitted to this procedure, the regional distribution, and the temporal evolution of its performance relevant research purposes. Thus, this study aimed to characterize patients hospitalized at the Brazilian Public Unified Health System who underwent tracheostomy in the period 2011-2020, according to sex, age, and macro-region of residence, with an analysis of the main causes of hospitalization and outcomes.

## METHODS

We conducted an ecological, observational study, with a quantitative approach and time series analysis. We studied the population residing in Brazil aged 20 years or older, who underwent a tracheostomy procedure in the period 2011 2020 in hospitals throughout the country, whose procedures were financed by the Unified Health System.

For the temporal analysis of the rate series, we defined as dependent variables the tracheostomies according to sex, age (20-29 years, 30-39 year, 40-49 years, 50-59 year, 60-69 years, 70-79 years, and 80+ years), major region of residence (North, Northeast, Southeast, South, and Midwest), cause classified by the ICD chapter (Chapter II, Chapter X, Chapter XIX, and others), and mortality and lethality of this procedure.

Data collection was performed through access to public databases of the Unified Health System (SUS), in particular the Hospital Information System (SIH) managed by DATASUS. We obtained the data of interest for this study with the aid of the TABWIN software. We used 3,240 files of reduced information on Hospital Admission Authorizations (AIH), corresponding to all hospitalizations financed by the SUS in all states of the federation and in the Federal District, in the period between January 2011 and December 2020.

After being extracted and tabulated, the data were exported to the MS-Excel software, which we used to organize the data in spreadsheets, as well as to perform calculations, adjustments, and to represent the results in the form of tables and graphs.

To calculate the rates and proportions, we used the ratio between the frequency of the study events and the population subject to the risk of occurrence of such events, whose result was multiplied by the constant of 100,000 or 100, as needed. We analyzed the results using the SPSS v.20.0 program (IBM, Armonk, New York, USA). For temporal trend analysis, we applied the Spearman’s correlation coefficient (ρ) and analysis of variance (ANOVA). Values of p<0.05 were considered significant.

Given that the present study is of an ecological type, based on secondary data in the public domain, without identification of the subjects, and that it used population aggregates as the unit of analysis, the submission to and appraisal by the Ethics Committees in Research Involving Human Beings was not carried out, in accordance with the terms of Resolution CNS 510/2016 (Article 1, Sole Paragraph, Items II, III and V).

. 

## RESULTS

According to the SIH/Datasus database, during the period studied (2011-2020), the SUS financed 172,456 tracheostomy procedures throughout the Brazilian territory in patients over 20 years of age.

Table 1 shows the tracheostomy rates by year and sex that occurred in Brazil in the study period. The evolution of annual rates in this period indicated a downward trend in tracheostomy performance in the hospitalized population in Brazil. This trend was greater in males (ρ=0.903, p=0.001). In the female population, the trend was not statistically significant (p=0.08), suggesting stability. The ratio of performing tracheostomies between the sexes (male/female) was 1.8:1.

**Table 1:** Tracheostomy performance rates (x100,000) according to sex and year of occurrence. Brazil, 2011-2020.

Year	Male	Female	Total
2011	16.83	8.34	12.42
2012	16.44	8.29	12.21
2013	16.73	8.93	12.68
2014	18.12	9.96	13.88
2015	15.46	8.80	12.00
2016	14.73	8.27	11.37
2017	15.35	8.49	11.79
2018	14.51	7.97	11.11
2019	13.99	7.92	10.84
2020	12.60	7.10	9.74
Average	15.48	8.41	11.80
Spearman ρ	-0.903	-0.661	-0.879
Beta	-0.859	-0.578	-0.786
p-value	0.001	0.08	0.007

Source: SIH/Datasus adapted by the author, 2021.

Table 2 shows the tracheostomy rates recorded in Brazil according to the year of occurrence and age group. There was a tendency of declining tracheostomy rates over the period studied. However, despite the general reduction in the number of tracheostomies performed over the years, there was an increased risk of performing this procedure in older age groups. Thus, when comparing the average tracheostomy rates performed in adults (6.6 procedures/100,000 inhabitants) with the average tracheostomy rates performed in the elderly (38.61 procedures/100,000 inhabitants), the elderly (60 years or older) had a 5.85 times greater risk of undergoing tracheostomy than adults (20-59 years).

**Table 2:** Tracheostomy performance rates (x100,000) according to age group and year of occurrence. Brazil, 2011-2020.

Year	Age							Total
	20-29	30 - 39	40 - 49	50 - 59	60 - 69	70 - 79	80 - +	
2011	3.71	4.22	7.36	15.58	27.05	45.47	50.52	11.56
2012	3.87	4.20	7.11	14.26	26.15	44.79	49.75	11.34
2013	3.76	4.39	7.14	14.17	26.12	45.41	57.19	11.67
2014	4.18	4.51	7.87	15.42	28.39	49.99	63.31	12.87
2015	3.54	3.94	6.18	12.82	24.53	43.45	55.85	11.09
2016	3.44	3.62	6.33	11.77	23.28	40.43	48.66	10.51
2017	3.15	3.66	5.97	12.40	24.36	42.07	50.19	10.85
2018	2.78	3.05	5.51	11.75	23.09	40.58	44.82	10.21
2019	2.49	2.88	5.30	11.62	21.91	35.47	40.06	9.62
2020	2.35	2.76	4.99	10.63	20.30	32.52	32.56	8.91
Average	3.33	3.72	6.38	13.04	24.52	42.02	49.29	10.86
Spearman ρ	-0.891	-.879**	-.903**	-.952**	-.915**	-.879**	-.733*	-.879**
Beta	-0.896	-0.916	-0.909	-0.912	-0.884	-0.837	-0.708	-0.814
p-value	0.000	0.000	0.000	0.000	0.001	0.003	0.022	0.004

Source: SIH/Datasus adapted by the author, 2021.

Table 3 shows the distribution of tracheostomy rates according to the year of occurrence, in large Brazilian regions, in the period 2011 2020. There was a trend towards stability in the rates in the period in the North and Northeast regions (ρ=0.152 and 0.455, p-value=0.431 and 0.329, respectively). In the other regions (Southeast, South, and Midwest) there was a tendency towards a reduction in tracheostomy rates (ρ=0.733, 0.879, and 0.636, p-value=0.022, 0.001, and 0.064, respectively). The South Region stood out for the strong downward trend observed (ρ= 0.879, p<0.001). It is also worth noting that the average rate observed in the South Region (24.17 procedures/100,000 inhabitants) was more than twice as high as the average Brazilian rate (11.8 procedures/100,000 inhabitants).

**Table 3:** Tracheostomy performance rates (x100,000) according to major Brazilian regions and year of occurrence. Brazil 2011-2020.

Year	North	Northeast	Southeast	South	Midwest	Total
2011	8,57	9,97	10,51	26,20	8,62	12,42
2012	10,34	9,97	8,96	27,06	11,52	12,21
2013	8,08	9,91	9,42	29,72	12,22	12,68
2014	9,57	12,33	10,04	30,17	13,72	13,88
2015	7,94	12,77	8,32	24,37	10,30	12,00
2016	10,33	12,64	6,65	23,96	10,61	11,37
2017	11,62	13,11	7,52	22,47	11,02	11,79
2018	10,33	12,64	7,38	21,09	8,57	11,11
2019	11,18	11,18	8,28	20,03	6,04	10,84
2020	8,04	10,11	7,96	16,66	7,06	9,74
Média	9,60	11,46	8,50	24,17	9,97	11,80
Spearman	0,152	0,455	0,733*	0,879**	0,636*	-,879**
Beta	0,281	0,345	-0,706	-0,864	-0,605	-0,786
p-valor	0,431	0,329	0,022	0,001	0,064	0,007

Source: SIH/Datasus adapted by the author, 2021.

Table 4 shows tracheostomy rates according to year of occurrence and causes of hospitalization, classified by chapters of the International Classification of Diseases - ICD-10. Neoplasms (Chapter II), diseases of the respiratory system (Chapter X), and those classified as other causes showed a strong downward trend in the period (ρ=0.952, 0.879, and 0.879, and p-value 0.001, 0.006, and 0.001, respectively). Oncological hospitalizations and other causes were statistically significant (p<0.001). The chapter that aggregated external causes (Chapter XIX) showed a trend towards stability in the period. The highest rates of tracheostomy procedures were related to hospitalizations for respiratory causes (Chapter X), the respiratory system being the main cause of tracheostomy in the last 10 years in Brazil, and responsible for 73% of tracheostomies performed in this time interval. In addition, the ICD that deals with neoplasms had an important reduction, of 20.74%, in the average number of tracheostomies performed in the last year studied, 2020.

**Table 4:** Tracheostomy rates classified by ICD chapters and other causes (x100,000) and by year of occurrence. Brazil, 2011-2020.

Year	Chapter II	Chapter X	Chapter XIX	Others	Total
2011	2.03	9.18	0.74	0.47	12.42
2012	1.94	9.03	0.75	0.49	12.21
2013	1.71	9.50	0.85	0.62	12.68
2014	1.59	10.60	1.08	0.60	13.88
2015	1.60	8.99	0.77	0.64	12.00
2016	1.55	8.37	0.70	0.76	11.37
2017	1.51	8.51	0.80	0.97	11.79
2018	1.48	7.93	0.81	0.88	11.11
2019	1.51	7.65	0.86	0.82	10.84
2020	1.35	6.75	0.79	0.84	9.74
Average	1.63	8.65	0.82	0.71	11.80
spearman	-.952**	-.879**	0.297	.879**	-.879**
Beta	-0.919	-0.794	0.028	0.885	-0.786
p-value	0.001	0.006	0.938	0.001	0.007

Source: SIH/Datasus adapted by the author, 2021. Chapter II: Neoplasms; Chapter X: Diseases of the Respiratory System; Chapter XIX: Injuries, Poisonings and Other Consequences of External Causes.

Table 5 brings the mortality and lethality rates by year of occurrence. Both mortality and lethality rates tended to decrease, with statistical significance. We also observed that tracheostomy was associated with a high mortality rate (28.57%). However, the case fatality rate decreased over the years (ρ=0.976, p<0.001).

**Tabela 5:** Taxas de morte e letalidade da traqueostomia (x100000) e por ano de ocorrência. Brasil, 2011-2020.

Ano	Mortalidade (x100.000)	Letalidade (%)
2011	3,99	32,09
2012	3,69	30,24
2013	3,78	29,78
2014	4,18	30,13
2015	3,47	28,90
2016	3,20	28,10
2017	3,18	27,01
2018	3,00	26,99
2019	2,77	25,55
2020	2,57	26,40
Total	3,36	28,57
Spearman	-,915**	-,976**
Beta	-0,914	-0,964
p-valor	<0,001	<0,001

Source: SIH/Datasus adapted by the author, 2021.

## DISCUSSION

Tracheostomy is a medical procedure widely used in prolonged mechanical ventilation^13^. The population eligible for its performance is critically ill patients admitted to the ICU^14^. For this reason, it is associated with high mortality and lethality rates, which was confirmed by our results, tracheostomy not being the direct cause of these unfavorable outcomes^15^. Despite the expressive lethality rates observed in Brazil, which demonstrates its association with poor outcomes, there was a trend towards a reduction in its frequency in the studied period. Possible explanations for this movement may be the reduction in mortality from acute respiratory distress syndrome (ARDS), and the development and incorporation of new mechanical ventilation techniques that occurred in the period^16^. Thus, the improvement in the prognosis of ARDS, one of the main causes of hospitalization leading to mechanical ventilation, positively influenced the lethality related to support procedures in this group of patients. Tracheostomy is a surgical procedure indicated in patients with critical clinical or surgical conditions, the mortality of patients with tracheostomy not being synonymous with mortality from tracheostomy. Corroborating this statement, a study carried out in São Paulo with the objective of comparing mortality between the indication and early or late performance of tracheostomy found no difference in mortality between the two procedures studied but associated the indication of tracheostomy with reduced mortality in the ICU studied^17^.

The frequency of tracheostomy procedures distributed by sex showed a great difference, males being responsible for 64.7% of these procedures performed in Brazil. Several international studies confirm this male prevalence. In Iran, men were responsible for 66.1% of tracheostomies^18^; in Havana, by 59.4%^19^; and in Nigeria, by 63%^20^. This prevalence can be explained by the main causes of hospitalization in men being diseases of the respiratory system^21^, which were the main cause of indication for the tracheostomy procedure in Brazil during the period studied.

As for the Brazilian large regions, the average rates of tracheostomy performance in the period studied in the South Region stood out for being twice as high as the average Brazilian rate. A similar study carried out in Europe indicated that, in high-income European countries, tracheostomy was more frequent when compared to middle-income countries^22^. The indication and performance of tracheostomies is strongly dependent on the existence and geographic distribution of intensive care beds, which in turn are concentrated in the South and Southeast regions of Brazil, explaining their higher frequency in such regions. Another explanation is that the South Region has a longer period of low temperatures when compared to the other Brazilian regions. According to Pajman et al., the incidence and mortality from sepsis of respiratory origin are strongly concentrated in the winter period^23^.

Our results also indicate that the elderly are 5.85 times more likely to undergo tracheostomy than adults. In line with these data, Dolin^2^4 states that the elderly have a greater chance of progressing to severe forms when affected by diseases of the respiratory system, as they have decreased lung compliance, respiratory muscle strength, cellular immunity, and response of B cells to new antigens. Se Hee Na et al. also confirmed that the elderly tend to display more severe conditions when admitted to the ICU^25^. Mark D Siegel^16^ also reports that the incidence of ARDS increases with age, reaching 306 individuals per 100,000 persons-year among patients aged 75 to 84 years.

The performance of tracheostomy procedures, as well as many other aspects of hospital care, has also been affected by the COVID-19 pandemic. One of these changes can be observed in the significant reduction, 20.74%, in the average number of tracheostomies performed in patients hospitalized by Chapter II of the ICD (cancer patients) conditions in relation to previous years, which can be explained by the obstacles to accessing the health system occurred since the beginning of the COVID-19 pandemic. With the system overloaded by the occupation of beds, the lack of professionals, and the shortage of medicines^26^ , there was a delay in cancer screenings and treatments in the last year studied^27^ ^,^ ^28^. Allied to this scenario, due to their own condition or the effect of the drugs in use, cancer patients became a risk group for severe COVID due to low immunity, which raised the requirements for the indication of hospital admissions in this population. The repressed demand observed because of the pandemic and the impact of COVID infection on general mortality were such that directly interfered even with broader indicators such as life expectancy, whose reduction was observed in most of the 29 countries studied by Aburto et al.^15^.

Surprisingly, among the effects of the COVID-19 pandemic, which was characterized by a significant increase in the number of patients who required mechanical ventilation, there was no increase in tracheostomy rates in the country in 2020. A possible explanation for the reduction in the tracheostomies frequency observed in 2020 may be its association with a greater risk of respiratory contamination for all health team members^29^ , including professionals who perform checking, suction, dressing change, and other post tracheostomy care^30^. Because of this, most protocols started to postpone tracheostomy in patients with COVID. According to Smith, it is now indicated after 21 days of intubation, and conditioned to negative COVID-19 tests. Anesi confirms that, despite not being a consensus, the procedure was always indicated after 10 days of intubation^31^.

The results found in this nationwide survey showed that tracheostomy is a procedure widely performed throughout the country, despite being more frequent in the South Region, a condition probably justified, on the demand side, by the higher prevalence of severe respiratory conditions and the higher proportion of elderly people, and facilitated, on the supply side, by the better structure of services, with a higher proportion of critical intensive care.

## CONCLUSION

The research carried out indicated that the prevalent sociodemographic profile of patients undergoing tracheostomy in Brazil in the period 2011 2020 was male, elderly, with emphasis on the age group of 80 years or older, residing in the Southern Brazilian region, having diseases of the respiratory system as primary causes for the procedure.

Despite the high mortality and lethality rates associated with hospitalizations that included tracheostomy procedures, the temporal trend of tracheostomy rates performed in Brazil was of reduction in the studied period, with greater emphasis on males.

The risk of undergoing tracheostomy grew in line with increasing age, and was concentrated in the older age groups.

The temporal trend of tracheostomy performance among the major Brazilian regions was stability in the North and Northeast, and reduction in the Southeast, South, and Midwest regions. As for the tracheostomy indications, hospitalizations associated with oncological and respiratory causes and those classified as “other causes” showed a downward trend in the period.

**Figure 1:**
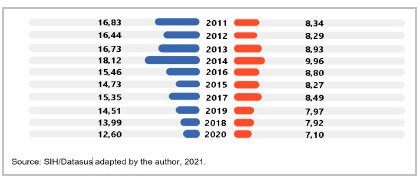
Tracheostomy performance rates (x100,000) according to sex and year. Brazil, 2011-2020.

**Figure 2:**
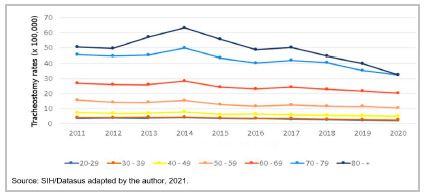
Tracheostomy performance rates (x100,000) according to age group and year. Brazil, 2011-2020.

**Figure 3:**
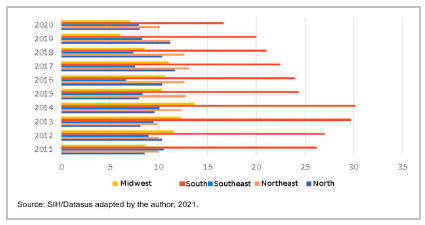
Tracheostomy performance rates (x100,000) according to major Brazilian regions and year. Brazil, 2011-2020.

**Figure 4:**
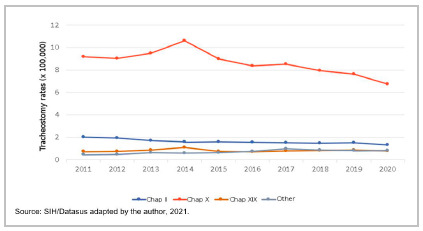
Tracheostomy rates classified by ICD chapters (x100,000) and year. Brazil, 2011-2020.

**Figure 5:**
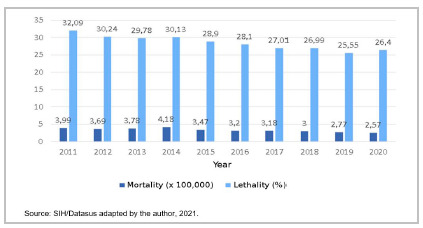
Tracheostomy death and lethality rates (x100,000) and by year of occurrence. Brazil, 2011-2020.
